# Propofol impairs specification of retinal cell types in zebrafish by inhibiting Zisp-mediated Noggin-1 palmitoylation and trafficking

**DOI:** 10.1186/s13287-021-02204-0

**Published:** 2021-03-20

**Authors:** Xiaoqing Fan, Haoran Yang, Lizhu Hu, Delong Wang, Ruiting Wang, Aijun Hao, Xueran Chen

**Affiliations:** 1 Department of Anesthesiology, Division of Life Sciences and Medicine, The First Affiliated Hospital of USTC, University of Science and Technology of China (USTC), No. 17, Lujiang Road, Hefei, 230001 Anhui China; 2 Department of Laboratory Medicine, Hefei Cancer Hospital, Chinese Academy of Sciences, No. 350, Shushan Hu Road, Hefei, 230031 Anhui China; 3 Anhui Province Key Laboratory of Medical Physics and Technology, Institute of Health and Medical Technology, Hefei Institutes of Physical Science, Chinese Academy of Sciences, No. 350, Shushan Hu Road, Hefei, 230031 Anhui China; 4 Key Laboratory for Experimental Teratology of Ministry of Education, Shandong Key Laboratory of Mental Disorders, Department of Anatomy and Histoembryology, School of Basic Medical Sciences, Cheeloo College of Medicine, Shandong University, No. 44, Wenhua Xi Road, Jinan, 250012 Shandong China

**Keywords:** Noggin, Palmitoylation, Propofol, Retina, Zebrafish, Zisp

## Abstract

**Background:**

Propofol can have adverse effects on developing neurons, leading to cognitive disorders, but the mechanism of such an effect remains elusive. Here, we aimed to investigate the effect of propofol on neuronal development in zebrafish and to identify the molecular mechanism(s) involved in this pathway.

**Methods:**

The effect of propofol on neuronal development was demonstrated by a series of in vitro and in vivo experiments. mRNA injections, whole-mount in situ hybridization and immunohistochemistry, quantitative real-time polymerase chain reaction, terminal deoxynucleotidyl transferase-mediated dUTP nick-end labeling, 5-ethynyl-2′-deoxyuridine labeling, co-immunoprecipitation, and acyl–biotin exchange labeling were used to identify the potential mechanisms of propofol-mediated zisp expression and determine its effect on the specification of retinal cell types.

**Results:**

Propofol impaired the specification of retinal cell types, thereby inhibiting neuronal and glial cell formation in retinas, mainly through the inhibition of Zisp expression. Furthermore, Zisp promoted the stabilization and secretion of a soluble form of the membrane-associated protein Noggin-1, a specific palmitoylation substrate.

**Conclusions:**

Propofol caused a severe phenotype during neuronal development in zebrafish. Our findings established a direct link between an anesthetic agent and protein palmitoylation in the regulation of neuronal development. This could be used to investigate the mechanisms via which the improper use of propofol might result in neuronal defects.

**Supplementary Information:**

The online version contains supplementary material available at 10.1186/s13287-021-02204-0.

## Background

Accumulating evidence from animal studies has shown that anesthetics can have adverse effects on developing neurons, leading to persistent cognitive dysfunction [1–3]. Developmental neurotoxicity induced by anesthetics includes neuronal apoptosis, neurodegenerative changes, neurogenesis and synaptogenesis, and brain circuit damage [4–6]. Propofol is one of the most commonly used intravenous anesthetics in pediatrics. It has been found that propofol, even at non-toxic doses, can cause neuronal death in the brains of infant mice and lead to brain damage in fetuses and newborns of non-human primates [7, 8]. Propofol can also change the dynamic balance of calcium, resulting in mitochondrial dysfunction, neuroinflammation, and dystrophin expression [9, 10]; however, the detailed mechanism for these actions is not clear.

Palmitoylation, a post-translational lipid modification [11, 12], occurs in various neuronal proteins that are involved in the formation of neuronal processes and the spine, including Gα [13], glutamate receptors [14], neural cell adhesion molecule [15], synaptosomal-associated protein, 25 kDa [16], and postsynaptic density protein-95 [17]. The zinc-finger, Asp-His-His-Cys (DHHC) domain-containing family of palmitoyl acyltransferases (PATs), has many members, all of which contain a cysteine-rich domain with a core DHHC motif that is essential for PAT activity [18, 19]. Genetic studies have shown that deficiency or dysfunction of PAT activity is associated with several neurological disorders. For example, in one patient with X-linked mental retardation, *DHHC15* transcript variants were absent [20]. *DHHC8* has been linked to schizophrenia in humans and neurophysiological deficits in mice [21, 22]. Recently, it has been reported that DHHC5 influences neuronal differentiation in cultured *DHHC5*-GT (gene-trapped) mouse neural stem cells [23].

Zebrafish are becoming more popular as a model organism for studying neuronal function and development because they develop fast and are transparent during the early embryonic stages, which allow the collection of large amounts of data and convenient gain-of-function and loss-of-function analyses [24, 25]. Here, we investigated the effect and molecular mechanism of propofol on zebrafish neural development. We report that propofol impaired the specification of retinal cell types via Zisp-mediated effects on protein palmitoylation and trafficking. Our findings establish a direct link between propofol and the regulation of neuronal development, suggesting that the improper use of propofol might result in defects in the neuronal system.

## Methods

### Zebrafish maintenance and treatment

All embryos were maintained at 28.5 °C and staged according to Kimmel et al. [25]. To prevent pigment formation, embryos were raised in 0.2 mM 1-phenyl-2-thiourea (Sigma) after the prim-5 stage. Zebrafish embryos (50 per well) were randomly exposed to propofol (Sigma-Aldrich) at defined doses (2.5, 5, or 7.5 μg/ml) in 5 ml fish water or control treatment (DMSO; 0.014%) by immersion from 10 to 48 postfertilization (hpf) in six-well plates.

### Constructs, cDNA cloning, and mutagenesis

Zebrafish full-coding *zisp* cDNA was isolated from a prim-5 total embryo cDNA library using the Clontech RACE cDNA synthesis and amplification kit (Invitrogen) with 5-CCCATCGATATGCCCAACAGCGTTGGAAAAAGAT-3′ forward and 5′-TCAGCTTTCGAA CACGGAGATCTCATATGTGGTTCCG-3′ reverse primers. Green fluorescent protein (GFP)-labeled Zisp was generated by inserting enhanced GFP (Clontech) at the N-terminus. Zisp ΔDHHC was generated by deleting the nucleotides encoding amino acids 104–145. Hemagglutinin (HA) fusion proteins of the wild type and mutant forms of Noggin-1 were generated by polymerase chain reaction (PCR) and subcloned into the XhoI and XbaI sites in frame with HA in a pcDNA3.0 plasmid. Cysteine-to-alanine mutant constructs were generated using the QuikChange Site-directed Mutagenesis Kit (Stratagene). All constructs were verified by DNA sequencing.

### mRNA injections

Zebrafish *zisp* mRNAs were transcribed in vitro from linearized pCS2+ constructs using an mMESSAGE kit (Ambion). For overexpression experiments, 80–200 pg mRNA was injected at the one- to two-cell stage.

### Antibodies

The following primary antibodies were used: mouse anti-α-acetylated-tubulin antibody (Santa Cruz Biotechnology; 1:1000), mouse anti-Islet-1 antibody (DSHB; 1:200), mouse anti-Zn-8 antibody (ZIRC; 1:200), rabbit anti-glial fibrillary acidic protein (Sigma; 1:500), mouse anti-Zpr-1 antibody (ZIRC; 1:200), mouse anti-HA antibody (Santa Cruz Biotechnology; 1:100), and mouse anti-GFP antibody (Molecular Probes; 1:200).

### Tissue sectioning and histology

Enucleated eyes were fixed in 4% paraformaldehyde overnight, dehydrated with series ethanol treatment, and embedded in paraffin. Hematoxylin-eosin staining and histology were performed as described previously [26] on 2–6-μm paraffin sections.

### Whole-mount in situ hybridization and immunohistochemistry

Whole-mount in situ hybridization and immunohistochemistry were performed as described previously [27, 28].

Sox2, Pax6.1, Islet-1, NeuroD4, and Crx mRNA probes labeled with digoxigenin (Roche, Basel, Switzerland) were synthesized from linearized template DNA by T7 RNA Polymerase (Roche) using in vitro transcription systems. DIG-labeled riboprobes (3 ng/μL) were hybridized to the samples overnight at 60 °C in hybridization buffer, then incubated overnight at 4 °C with an anti-DIG-AP antibody. After washing and equilibrating in alkaline phosphatase (NTMT) buffer, embryos were stained with 4-nitro blue tetrazolium (NBT; Roche), 5-bromo-4chloro-3-indolyl-phosphate, and 4-toluidine salt (BCIP; Roche) in NTMT buffer. A stop solution (phosphate-buffered saline (PBS) pH 5.5, 1 mM EDTA) was used to end the staining reaction, and embryos were then placed in 40% glycerol for imaging. Six embryos were analyzed per time point for each probe.

Thereafter, 5-μm paraffin sections were deparaffinized and blocked in 3% bovine serum albumin (BSA, w/v) for 30 min at room temperature (RT) and incubated in primary antibodies overnight at 4 °C. After washing twice in PBS, sections were incubated with the appropriate FITC-conjugated anti-rabbit IgG or RITC-conjugated anti-mouse IgG at RT for 2 h. Counterstaining with DAPI was performed to visualize the nuclei. The sections were then washed three times with PBS and covered with VECTASHIELD® mounting medium (Vector Laboratories) before placing the coverslips. At least six sections were analyzed per slide and per antibody.

### Terminal deoxynucleotidyl transferase dUTP nick-end labeling (TUNEL) assay

TUNEL assays were performed with an in situ cell death detection kit (Roche). Briefly, the fixed fish embryo sections were incubated for 15 min in the permeabilization solution, on ice. The samples were then labeled with the TUNEL reaction mixture for 1 h at 37 °C in the dark, prior to being washed three times with PBS for 10 min and kept at 4 °C.

### 5-Ethynyl-2′-deoxyuridine (EdU) labeling of proliferating cells

EdU incorporation and detection were performed as described previously [29]. Larvae were immersed in 400 μM EdU. Fish were then sacrificed and fixed in 4% PFA. EdU (Click-iT® Labeling Technologies, Life Technologies) incorporation was detected on paraffin sections with Alexa Fluor 488 before proceeding to IHC detection.

### Quantitative real-time PCR

The total RNA was isolated from embryos at various developmental stages using TRIzol reagent (Invitrogen) according to the manufacturer’s protocol. First-strand cDNA was synthesized from 2 μg total RNA using RevertAid™ First-Strand cDNA Synthesis Kit (Fermentas) and a random or oligo-dT primer. Quantitative PCR (qPCR) was performed using iQSybr Green Supermix (Bio-Rad). cDNA levels were determined using relative quantification and normalized to β-actin. The qPCR conditions were as follows: pre-denaturation at 95 °C for 10 s, followed by 40 cycles of amplification at 95 °C for 10 s, 55 °C for 10 s, and 72 °C for 15 s. Each sample was run in triplicate. A melting-curve analysis was performed to ensure the specificity of the products.

### Cell culture, transfection, and immunofluorescence

Human retinal pigment epithelium-1 (RPE1) cells gifted from Pro. Zhiyou Fang (Hefei Institutes of Physical Science, Chinese Academy of Sciences, Hefei) and cultured as described previously [30]. For transient expression, RPE1 cells were transfected with Lipofectamine 2000 (Invitrogen) according to the manufacturer’s protocol. At 24 h post-transfection, cells were processed as described for each experiment. Cells were fixed in 4% paraformaldehyde in PBS (comprising 20 mM sodium dihydrogen phosphate, 0.9% sodium chloride [NaCl], pH 7.4), permeabilized with 0.3% Triton X-100 (v/v), and labeled with primary antibodies for 1 h at room temperature followed by incubation with appropriate secondary antibodies conjugated to Alexa 488 or Alexa 568.

### Immunoprecipitation and acyl–biotin exchange (ABE) labeling method

Cells were lysed for 30 min at 4 °C in ice-cold buffer (50 mM Tris hydrochloride, pH 7.5; 150 mM NaCl; 1% Triton; 1 mg/ml protease inhibitor cocktail; and 0.25 mg/ml phenylmethylsulfonyl fluoride) and centrifuged for 15 min at 14,000 rpm. Then, the lysates were incubated for 1 h at 4 °C with protein A + G agarose (Beyotime) containing 2 μg anti-GFP antibody. To determine the level of palmitoylation, the ABE assay was performed as described previously [31]. Briefly, the immunoprecipitated beads were incubated for 1 h at 4 °C with washing buffer (50 mM Tris, pH 7.4; 5 mM ethylenediaminetetraacetic acid; 150 mM NaCl; and 1% Triton X-100) supplemented with 50 mM *N*-ethylmaleimide. Next, samples were incubated with 1 M hydroxylamine (pH 7.4) for 1 h at room temperature. To label reactive cysteine residues, the beads were incubated with 0.5 μM 1-biotinamido-4-(4′-[maleimidoethylcyclohexane]-carboxamido) butane (pH 6.2) for 1 h at 4 °C. Samples were analyzed by sodium dodecyl sulfate-polyacrylamide gel electrophoresis and immunoblotting.

### Imaging and statistical analyses

Images were acquired using a stereomicroscope (Olympus SZX16) for in situ hybridizations, or a confocal laser scanning microscope (Leica DMIRE2) and a fluorescence microscope (Olympus IX71) for immunofluorescence imaging. Images were adjusted for brightness and contrast using Image-Pro Plus 6.0. Statistical analysis was performed with Student’s *t* test. All bar graphs are plotted as the means ± standard errors of the mean.

## Results

### Propofol impairs retinal growth and function in zebrafish

**Fig. 1:**
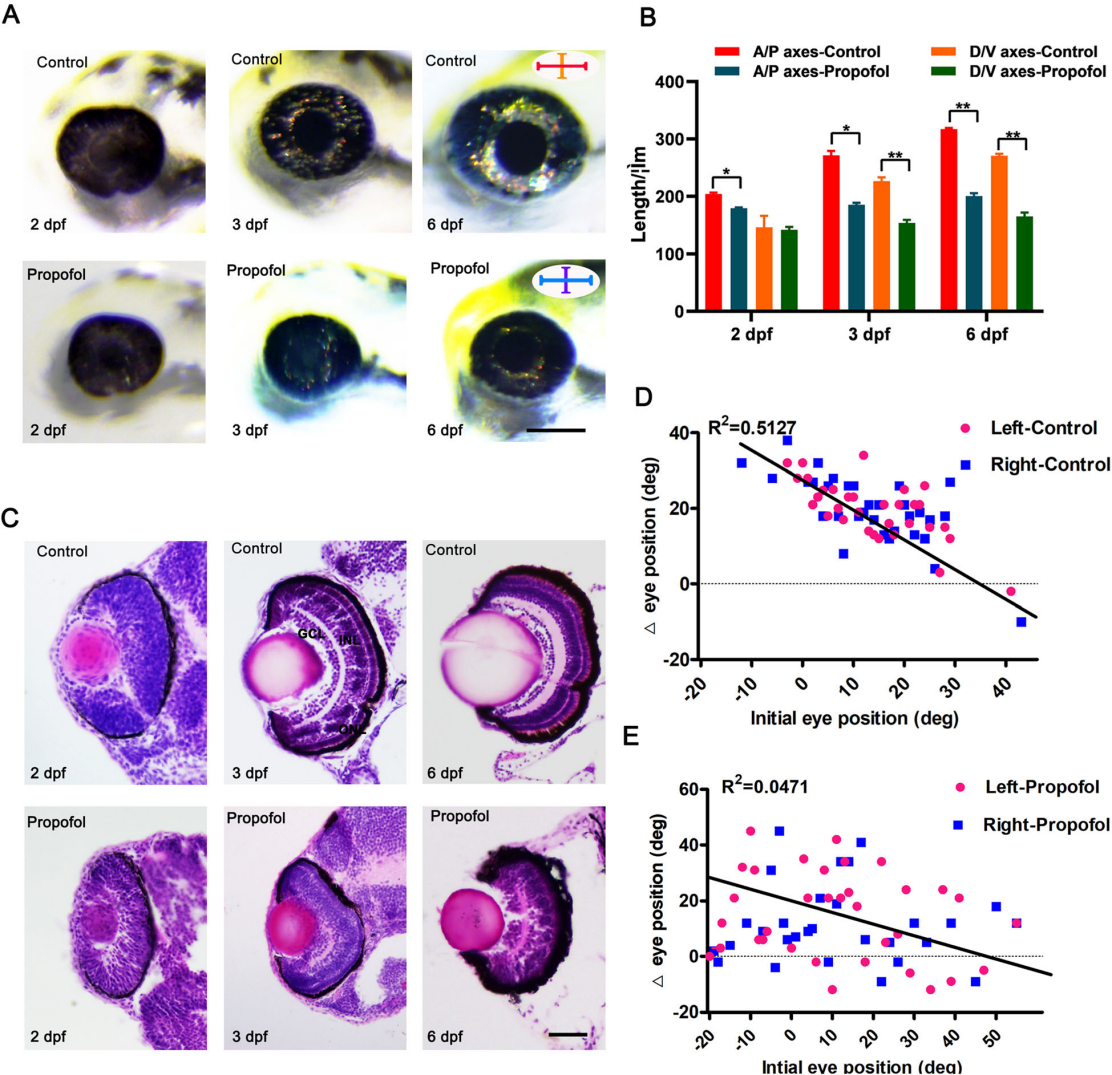
Propofol treatment results in microphthalmia and defects in retinal lamination and function. **a** Compared with control embryos, propofol-treated embryos (5 μg/ml propofol) at 2, 3, and 6 days postfertilization (dpf) display a reduction in eye size. **b** Measurements of absolute eye size along the anteroposterior (A/P) and dorsoventral (D/V) axes at 2, 3, and 6 dpf. *n* = 12. Error bars indicate the standard error of the mean. **c** Transverse histological sections of retinas of control and propofol-treated embryos (5 μg/ml propofol) at 2, 3, and 6 dpf. Eyes of propofol-treated embryos (5 μg/ml propofol) are smaller than control eyes, and the ganglion cell layer, inner nuclear layer, and outer nuclear layer are not morphologically identifiable in the retinas of propofol-treated embryos at 3 dpf. The dorsal side is up in all images. *n* = 7. **d**, **e** In response to prey, the extent of eye rotation is linearly associated with the initial position of the eye in control larvae at 6 dpf; in propofol-treated larvae (5 μg/ml propofol), there is no convergent eye movement. *n* = 35. **a** Scale bar, 100 μm. **c** Scale bar, 35 μm

To investigate the effect of propofol on zebrafish development, zebrafish embryos were placed in an anesthetic water bath containing 2.5, 5, or 7.5 μg/ml propofol. Treatment with propofol reduced eye size at 2 dpf (Fig. 1a, b) in a concentration-dependent manner (83.25 ± 8.1% for 2.5 μg/ml propofol, 90.12 ± 7.2% for 5 μg/ml propofol, and 92.67 ± 12.2% for 7.5 μg/ml propofol). At 3 dpf and 6 dpf, the retinas of propofol-treated zebrafish embryos had grown substantially, although the eyes remained smaller than those of controls. Based on the low mortality and relatively high microphthalmia observed in zebrafish treated with 5 μg/ml propofol, the remainder of the experiments were performed on this group of zebrafish.

Next, to clarify the effect of propofol on retinal development, we examined a series of retinal sections from propofol-treated zebrafish embryos (Fig. 1c). Histological analysis of control retinas at 2 dpf revealed well-laminated retinas with morphologically distinct ganglion cell layer (GCL), inner nuclear layer (INL), and outer nuclear layer (ONL) structures at 3 dpf and 6 dpf. In contrast, retinas from propofol-treated zebrafish embryos were poorly laminated at 2 dpf and 3 dpf, and neural layers were significantly underdeveloped, with most cells having a progenitor-like appearance; at 6 dpf, the GCL could be observed, although the INL and ONL were not discernible.

To investigate the effect of propofol on retinal function, we compared the initial response of larval fish to their prey between control and propofol-treated larvae [32]. At 6 dpf, the control larvae responded to paramecia with convergent eye movements, and a high vergence angle was maintained for the duration of the hunting routine (Additional file 1). Accordingly, the extent of eye rotation during convergence was linearly associated with the initial position of the eye in the control larvae (Fig. 1d). However, propofol-treated larvae exhibited abnormal behavior and lacked convergent eye movements in response to paramecia (Additional file 2), yet their motility was unaffected (Fig. 1e).

### Propofol impairs neuronal and glial differentiation in the retina

**Fig. 2:**
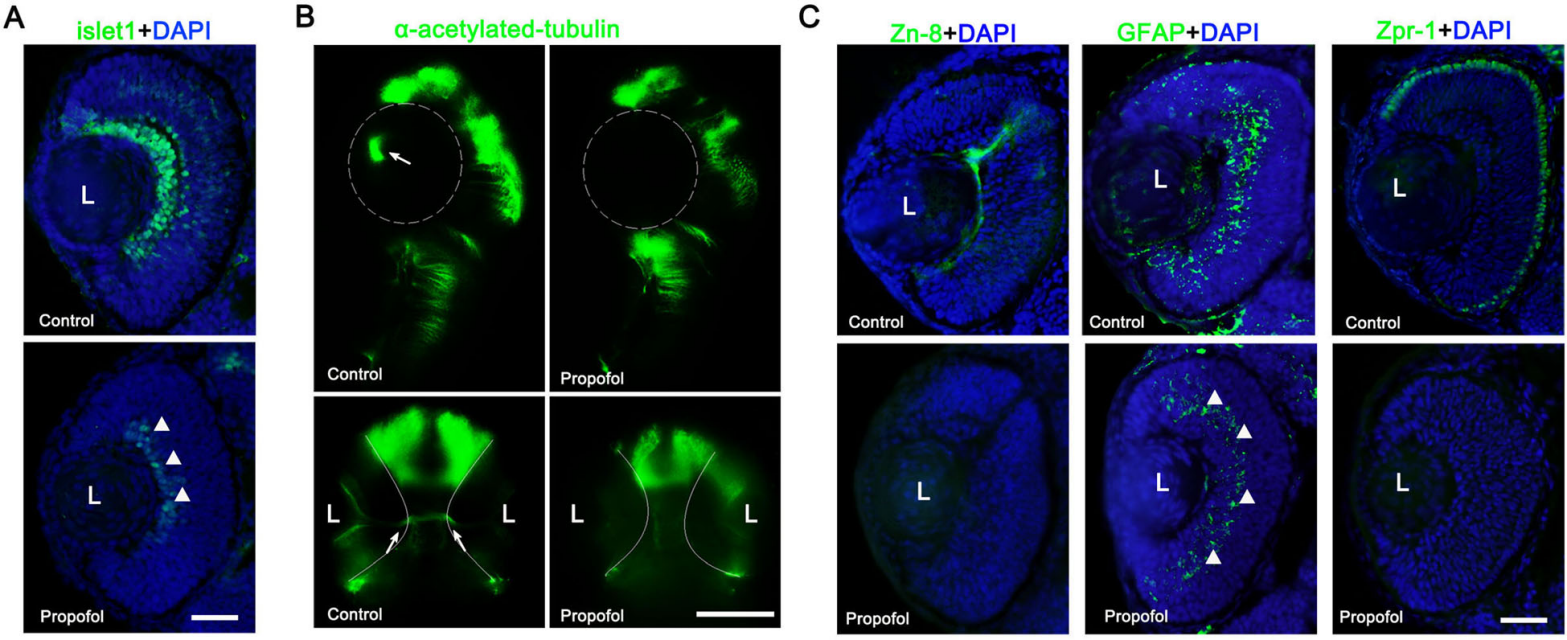
Propofol impairs the differentiation of retinal neurons and glia. Immunohistochemical analysis of markers (green) of neuronal and glial differentiation at 3 d postfertilization (dpf) (**a**, **c**) and 2 dpf (**b**). DNA, blue. **a**, **b** Although the retinas of propofol-treated embryos (5 μg/ml propofol) contain an occasional ganglion cell (**a**, arrowheads), optic axon tracts are absent (**b**). L, lens. The dotted line indicates the area of the eye. Arrows indicated optic axon tracts. **c** Ganglion cells (zn-8), Müller cells (GFAP), and red/green cones (zpr-1). With the exception of Müller cells (arrowheads), all late-differentiated cell types are absent. *n* = 5. Scale bar, 75 μm

As we mentioned above, there were no morphologically distinct GCL, INL, and ONL in the retinas of propofol-treated embryos, suggesting that propofol might impair neuronal and glial arrangement into a normal laminar organization. To test whether impaired neuronal and glial formation contributed to neuronal layer disruption in propofol-treated embryos, we compared the retinal expression of immunohistochemical markers of differentiated neurons and glia between control and propofol-treated embryos (Fig. 2). Islet-1, an early marker of neuronal differentiation, was found in the ganglion cells, amacrine cells, bipolar cells, and horizontal cells of control retinas. Retinas of propofol-treated embryos had small patches of Islet-1-positive cells and no optic axonal tracts (Fig. 2a, b), indicating the absence of neurite growth and guidance in the retinal ganglion cell. Several markers for later differentiation were used to test the presence of individual cell types. With the exception of an occasional Müller glial cell, the majority of cell types had not differentiated in the retinas of propofol-treated embryos (Fig. 2c). These results suggest that laminar organization of the neuronal layer was impaired in the retinas of propofol-treated embryos due to impaired neuronal and glial differentiation.

### Propofol does not affect the specification of retinal progenitor cell identity

During retinogenesis, retinal progenitor cells (retinoblasts) proliferate (1). Then, beginning with the GCL and proceeding outward to the INL and ONL, they follow paths to distinct cell fates and exit the cell cycle following a terminal mitosis (2). Next, they begin to express markers of terminal differentiation (3). Finally, they undergo neuronal or glial morphogenesis (4) [33, 34].

Retinas of propofol-treated embryos lacked morphologically identifiable neurons and glia. Moreover, these cells (with the exception of some retinal ganglion cells and Müller glia) did not express terminal differentiation markers, indicating that propofol might affect retinogenesis upstream of steps (3) and (4) described above. Therefore, the loss of neurons and glia in the retinas of propofol-treated embryos could be due to a change in retinal progenitor cell or retinal cell type specification.

**Fig. 3:**
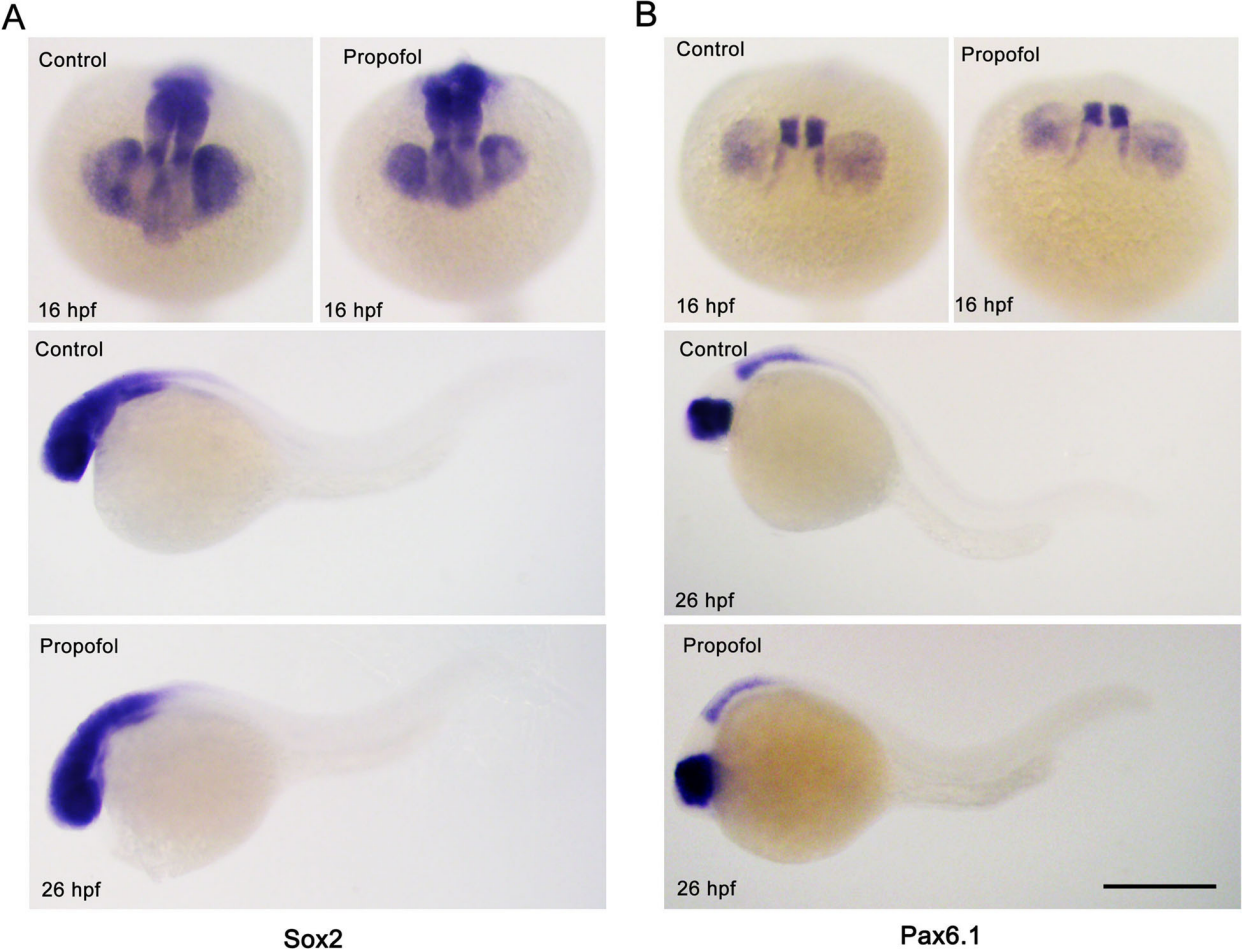
Propofol does not affect the specification of retinal progenitor cell identity. **a**, **b** The expression domains of both control and propofol-treated embryos (5 μg/ml propofol) at 16 and 26 h postfertilization (hpf) are shown for *sox2* (**a**) and *pax6.1* (**b**). The expression patterns of these progenitor identity markers are normal compared with those of control embryos. *n* = 18. Scale bar, 100 μm

To assess step (1) of retinogenesis (i.e., the specification of retinal progenitor cell identity), we analyzed the expression of retinal progenitor cell identity markers (*pax6a* and *sox2*) at the optic vesicle stage (16 hpf) and the optic cup stage (26 hpf) (Fig. 3). These markers were expressed normally, indicating that propofol did not affect the specification of retinal progenitor cell identity.

### Propofol affects the specification of retinal cell types

**Fig. 4:**
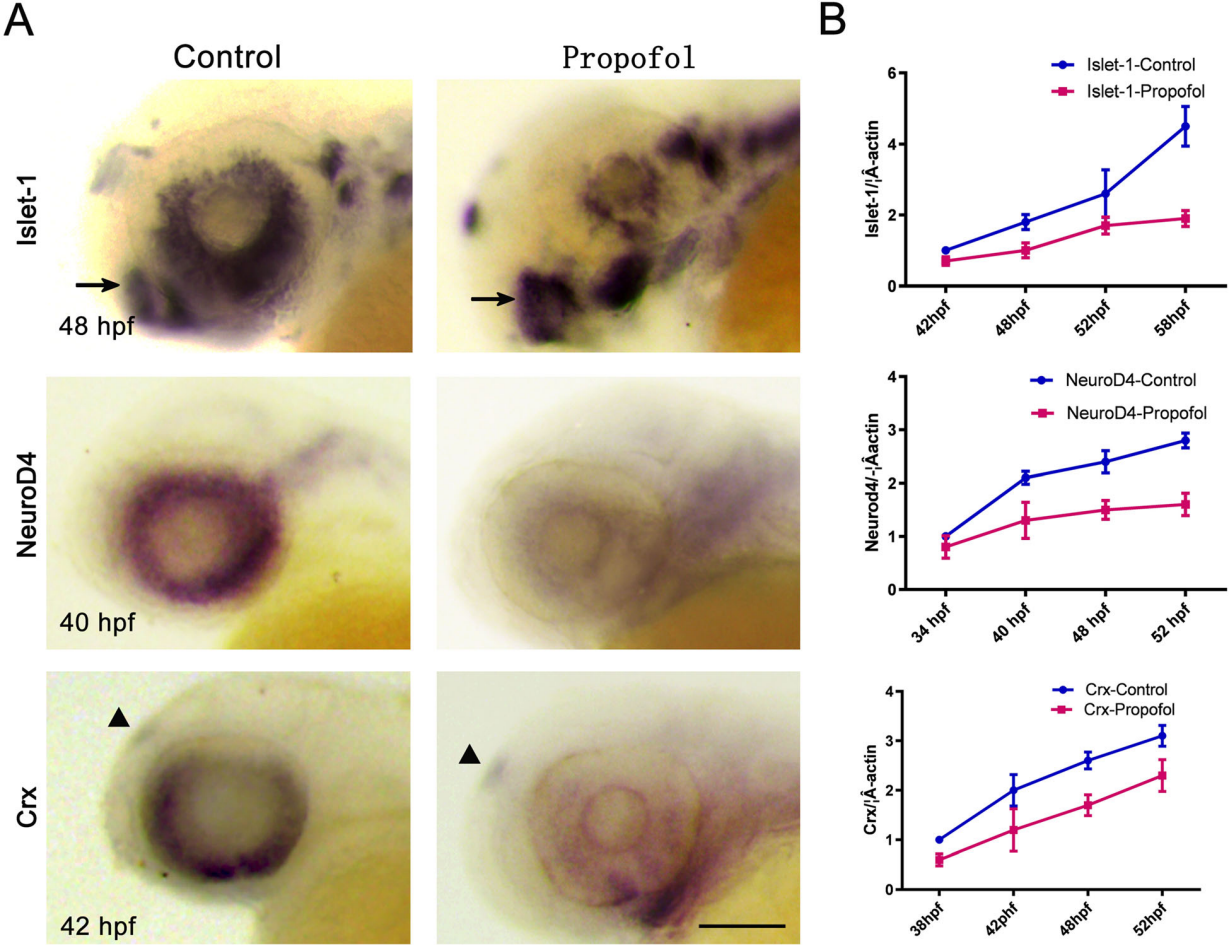
Propofol impairs the specification of retinal cell types. **a**, **b** Whole-mount in situ hybridization (**a**) and real-time quantitative polymerase chain reaction (**b**) were performed to detect the indicated genes (*isl1*, *neurod4*, and *crx*) at the indicated time points in control and propofol-treated embryos (5 μg/ml propofol). Expressions in the brain (*isl1*) and pineal body (*crx*) are indicated by arrows. **a** Lateral views. *n* = 21. Scale bar, 75 μm

To test the third possibility (i.e., whether propofol affected the specification of retinal cell types), we first analyzed the expression of specification markers using whole-mount in-situ hybridization (Fig. 4a) and real-time qPCR (Fig. 4b), including basic helix-loop-helix (bHLH) factor *neurod4* and homeobox factors *isl1* and *crx*. We found that propofol reduced the expression of these markers, suggesting that it impaired the specification of retinal cell types at an early time point. In control retinas, *neurod4* expression was confined to a ring-shaped area in the INL at 40 hpf; however, in the retinas of propofol-treated embryos, *neurod4* expression was reduced and limited to a much smaller patch of cells. In control retinas, *crx* was expressed in the presumptive ONL at 42 hpf; however, propofol-treated embryos lacked detectable *crx* expression. At 48 hpf, *isl1* expression was weak and confined mainly to the GCL; *isl1* signals were detected in the retinas of propofol-treated embryos. Expression of markers in the brain (*isl1*) and pineal body (*crx*) appeared relatively unaffected.

**Fig. 5:**
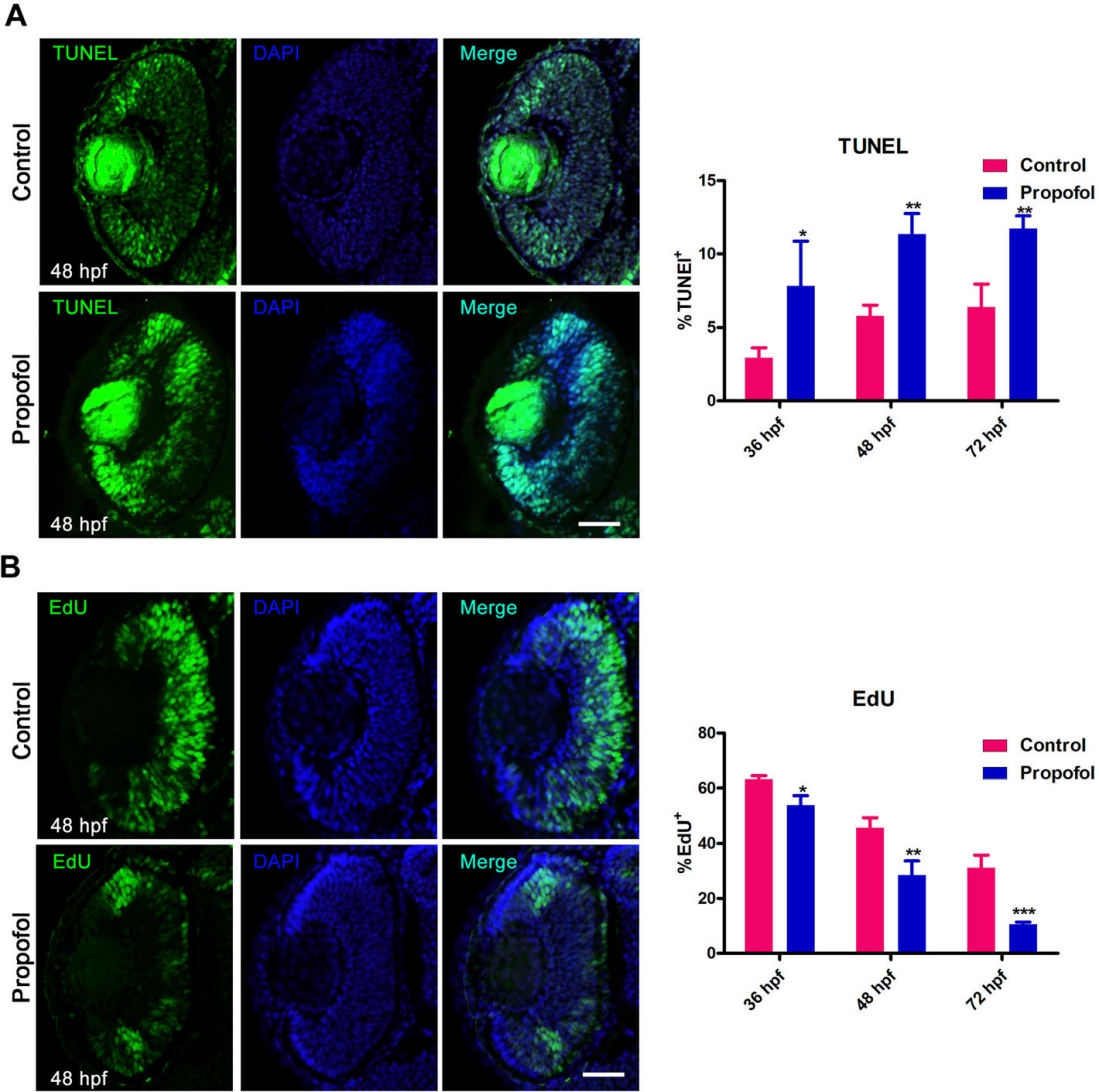
Propofol increases cell death and decreases the number of S-phase cells in retinas. **a** The number of terminal deoxynucleotidyl transferase dUTP nick-end labeling-positive cells (green) in the retinas of propofol-treated embryos (5 μg/ml propofol) at 36, 48, and 72 h postfertilization (hpf) is increased compared with that in controls. DNA, blue. *n* = 4. Scale bar, 25 μm. **b** EdU exposure at 36, 48, and 72 hpf decreases the proportion and mislocalization of S-phase cells in the retinas of propofol-treated embryos (5 μg/ml propofol) compared with control retinas. Error bars indicate the SEM. *, *p* < 0.05 and **, *p* < 0.01. *n* = 4. Scale bar, 18 μm

bHLH and homeobox factors are known to drive proliferative progenitor cells out of the cell cycle and stimulate their differentiation [34, 35]. First, we used the TUNEL assay to analyze the number of apoptotic cells in the retinas of control and propofol-treated embryos at 36 hpf, 48 hpf, and 72 hpf (Fig. 5a and Additional file 3a). The number of TUNEL-positive cells was higher in the retinas of propofol-treated embryos than in control retinas at any given time point, indicating an increase in cell death.

Next, we used a series of EdU incorporation assays to examine how retinoblasts exit the cell cycle. The percentage of EdU-positive cells was significantly lower in the retinas of propofol-treated embryos than in control retinas during and after the specification of retinal cell types (Fig. 5b and Additional file 3b). At 36 hpf (the initial stage of specification), the retinas of propofol-treated embryos contained 51.7% fewer EdU-positive cells than control retinas (64.3%). At 48 hpf (the intermediary stage of specification), the retinas of propofol-treated embryos contained 28.9% of EdU-positive cells, limited to the peripheral retina and the ciliary marginal zones; control retinas contained 46.2% of EdU-positive cells, spread throughout the central retina. At 72 hpf (the end stage of specification), only 8.1% of EdU-positive cells were found in the retinas of propofol-treated embryos, whereas 27.2% of control retinal cells remained in the S-phase.

Taken together, these data supported the third hypothesis that laminar organization of neuronal layer would be impaired due to alterations in the specification of retinal cell types. Indeed, in propofol-treated embryos, the specification of retinal cell types was inhibited because retinoblasts exited the cell cycle too early, i.e., cell cycle progression was compromised.

### Propofol impairs retinal development via inhibition of *zisp* expression

**Fig. 6:**
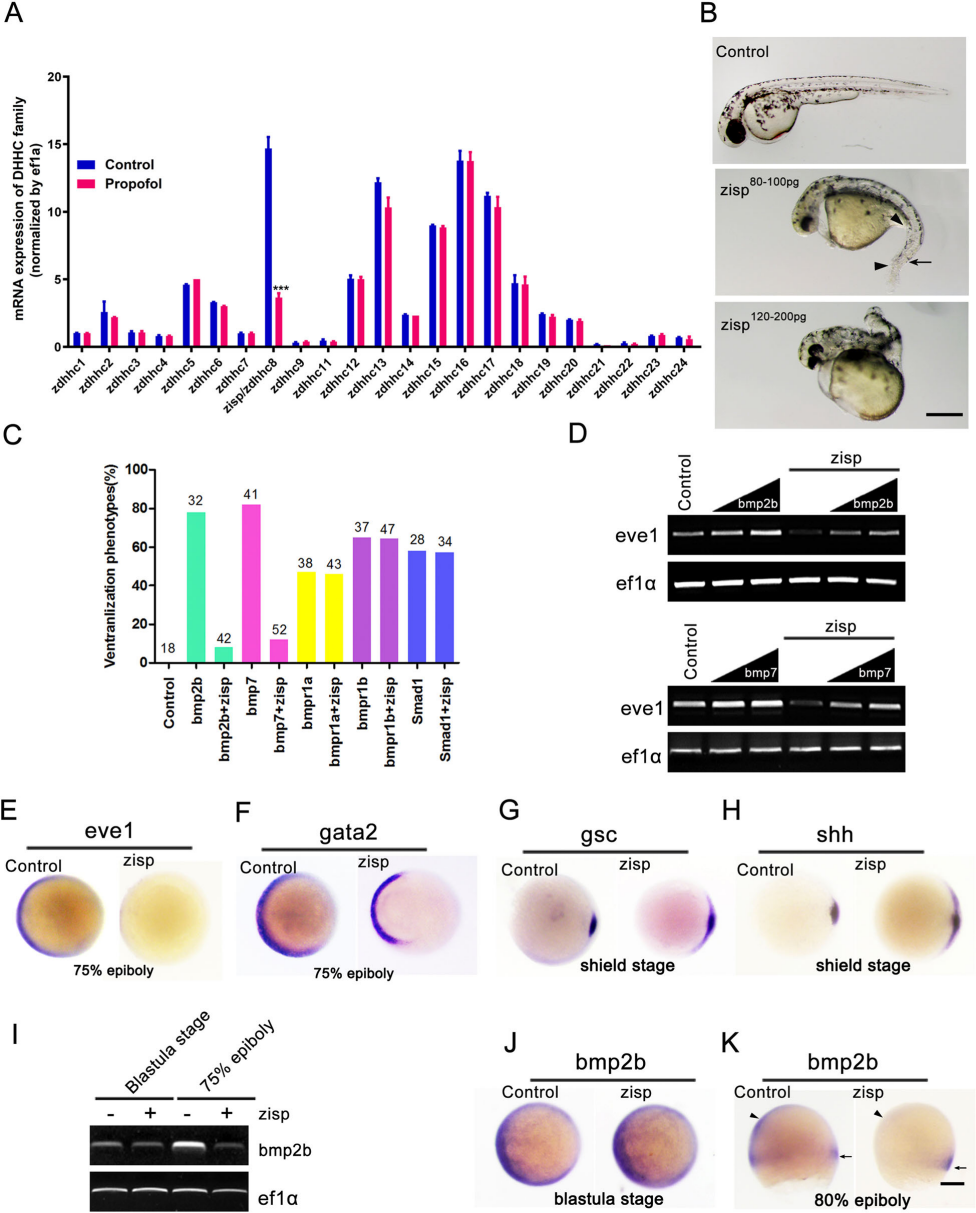
Zisp overexpression mimics noggin overexpression activity and acts upstream of the bone morphogenetic protein (BMP) receptor. **a** Quantitative reverse transcription polymerase chain reaction (qRT-PCR) analysis of mRNA levels of 24 known palmitoyl acyltransferases in propofol-treated retinas (5 μg/ml propofol). *Ef1a* was used as loading control. *n* = 3. **b** Injection of *zisp* mRNA induced dorsalization phenotypes, including an up-turned tail (arrow), a reduction of tail fin (arrowheads), and a reduction of tail and trunk. *n* = 13. Scale bar, 15 mm. **c** Zisp inhibited ventralization phenotypes by BMP ligands (*bmp2b* and *bmp7*) but not by BMP receptors (*bmpr1a* and *bmpr1b*) or Smad1. Numbers indicate the number of embryos scored. *n* = 3. **d** Zisp inhibited *bmp2b*- and *bmp7*-induced *eve1* expression. *ef1α* was used as an RT-PCR control. *n* = 3. **e–h** Following *zisp* overexpression, the *eve1* and *gata2* expression domains are greatly reduced (**e**, **f**), whereas the *shh* and *gsc* territories appear to have expanded (**g**, **h**). *n* = 17. **i–k** Following *zisp* overexpression, *bmp2b* expression is still present at the blastula stage (**j**), whereas it is lost in the ventral blastoderm (arrowheads) and maintained in the marginal zone (arrows) (**k**). **e–h**, **j** Animal pole views. **k** Lateral views. Scale bar, 75 μm

DHHC family-mediated palmitoylation markedly affects neural development, and deficiency or dysfunction of PAT activity is associated with several neurological disorders. We used quantitative reverse transcription (qRT)-PCR to analyze the expression of all members of the DHHC family in the retinas of propofol-treated embryos (Fig. 6a). We observed a significant decrease in *zisp*/*zdhhc8* expression levels after treatment with propofol. These results indicated that propofol impaired the specification of retinal cell types possibly via inhibition of *zisp* expression.

Notably, *zisp* transcripts were hardly detected before the bud stage (10 hpf) based on whole-mount in situ hybridization (Additional file 4a and 4b). At the 14-somite stage (16 hpf), in addition to the somitic expression, *zisp* transcripts were found in the optic vesicle (Additional file 4c and 4d) [36]. At 24 hpf, *zisp* was detected in the retina and lens (Additional file 4e). In the retina, *zisp* expression was restricted to the INL at 32 hpf (Additional file 4f) and 48 hpf and gradually decreased thereafter (Additional file 3g) until it disappeared at approximately 60 hpf. RT-PCR analysis revealed that *zisp* was maternally expressed in zebrafish (Additional file 4h).

There is a diverse network of intrinsic signaling pathways [37] and transcription factors [38] determining the normal specification and differentiation of multiple cell types during retinogenesis. To investigate the possible mechanism via which Zisp might be involved in retinogenesis, we overexpressed zebrafish Zisp in wild-type embryos at the one-cell stage (Fig. 6b). All embryos injected with 80–100 pg of zebrafish *zisp* mRNA showed a dorsalization phenotype characterized by an up-turned tail and a lack of the ventral fin, which was reminiscent of bone morphogenetic protein (BMP) signaling mutants [39]. Increasing the dose of *zisp* mRNA (120–200 pg) caused a stronger phenotype with further reduction of the tail and trunk in 68% of the injected embryos. Indeed, Zisp antagonized BMP function and inhibited ventralization phenotypes and *eve1* induction (a BMP target gene) by BMP ligands (*bmp2b* and *bmp7*) but not by BMP receptors (*bmpr1a* and *bmpr1b*) or Smad1 (a BMP downstream component) (Fig. 6c and d). Thus, Zisp likely acted upstream of BMP receptors or BMP signaling.

### Zisp promotes Noggin-1 secretion and stability

Early embryos were further analyzed using in situ hybridization and RT-PCR. Following Zisp overexpression, *eve1* and *gata2* (another BMP target gene) expressions were strongly reduced or even abolished (Fig. 6e and f), whereas *shh* and *gsc* (a homeobox gene) extended all around the margin (Fig. 6g and h). Notably, although *bmp2b* expression was unaffected at the early blastula stages, it was lost in the ventral blastoderm at later mid-gastrula stages in Zisp-overexpressing embryos (Fig. 6i, j). This pattern is similar to the biological activity of Noggin [40], indicating that Zisp might regulate Noggin.

**Fig. 7:**
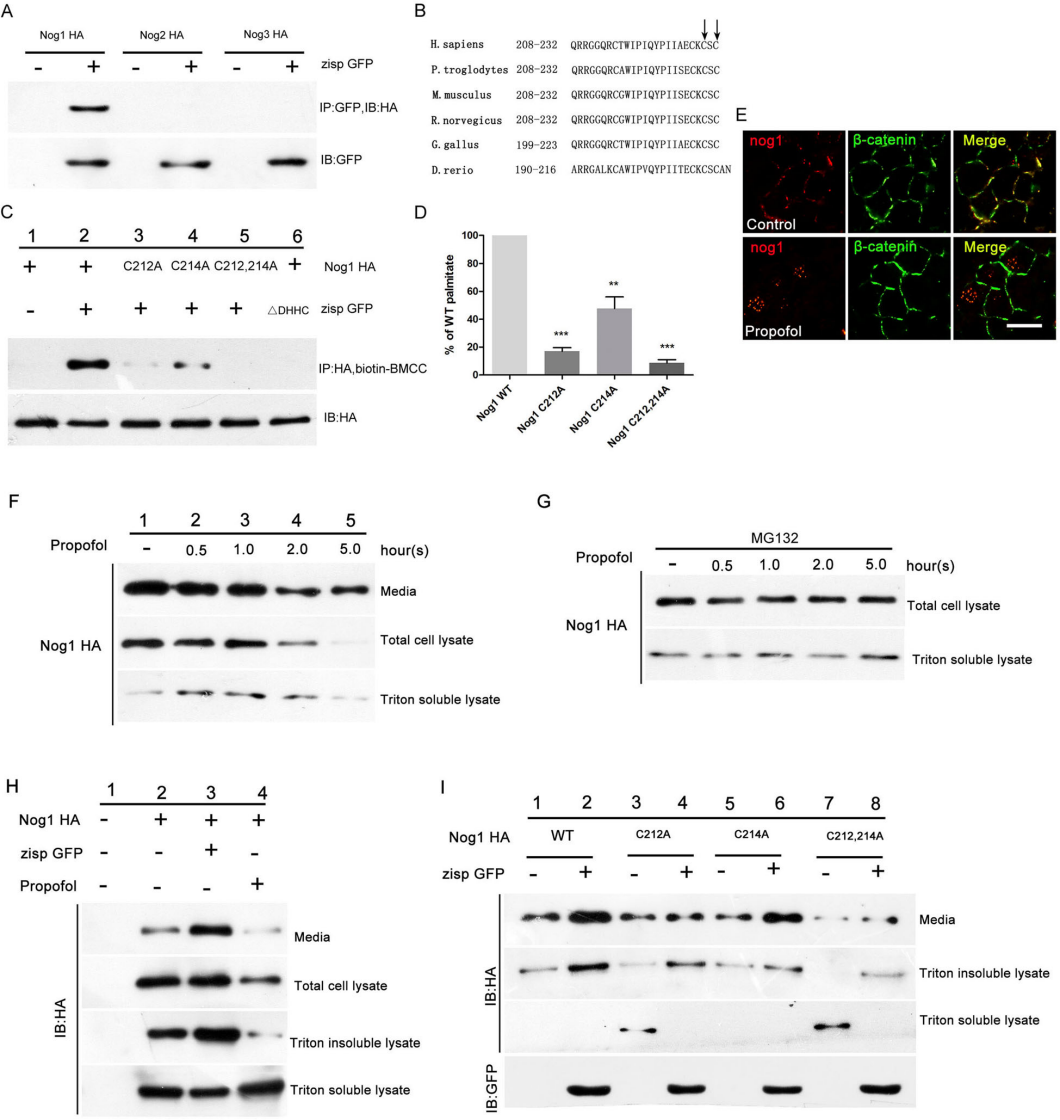
Palmitoylation increases the secretion and activity of Noggin-1. **a** Noggin-1 binds to Zisp. Embryos were co-transfected with green fluorescent protein (GFP)-tagged *zisp* and hemagglutinin (HA)-tagged *nog* (*nog1*, *nog2*, and *nog3*). Immunoprecipitation with anti-HA was performed, followed by western blotting for GFP. Zisp significantly enhanced Noggin-1 expression, while Noggin-2 and Noggin-3 were not detected. *n* = 3. **b** Sequence alignment of Noggin-1 proteins. The region containing residues 208–232 of zebrafish Zisp is aligned to that of other species. The arrows show the potential palmitoylation sites near the C-terminus. **c**, **d** Zisp induced palmitoylation of wild-type HA-tagged Noggin-1 but not of the mutant form of Noggin-1. However, Zisp △ Asp-His-His-Cys failed to palmitoylate HA-tagged Noggin-1. Values were first normalized with respect to the expression levels, and then, the expression of mutant forms was normalized to that of the wild-type proteins (**d**). *n* = 3. Error bars indicate the standard error of the mean. **p < 0.01 and ***p < 0.001. **e** Expression of Noggin-1 in the retinas of propofol-treated zebrafish (5 μg/ml propofol) was analyzed by immunofluorescence staining at 32 h postfertilization. The membranes were labeled with an anti-β-catenin antibody. *n* = 3. Scale bar, 100 μm. **f** Secretion of Noggin-1 was blocked by propofol. *n* = 3. **g** The proteasome inhibitor MG132 stabilized intracellular Noggin-1 (total cell lysate) and Noggin-1 in the soluble fraction (Triton-soluble lysate). *n* = 3. **h** GFP-labeled Zisp increased Noggin-1 secretion into the medium, while propofol blocked membrane targeting and secretion of transfected *nog1*. *n* = 3. **i** Cysteines 212 and 214 mediate part of Zisp activity. After co-transfection of *zisp* and *nog1* (wild-type or mutant) constructs into RPE1 cells, mutation of the cysteine residues reduced the basal level of Noggin-1 secretion into the medium. *n* = 3

Zebrafish possess four *nog* paralogs: *nog1*, *nog2*, *nog3*, and *nog4* [40]. To investigate whether Zisp regulates Noggin and, if so, to determine which Noggin protein is regulated, we co-transfected GFP-tagged Zisp and HA-tagged Noggin in RPE1 cells. We found that Zisp significantly enhanced Noggin-1 expression, while Noggin-2 and Noggin-3 were not detected (Fig. 7a).

Structurally, Zisp is predicted to have four hydrophobic segments (probably transmembrane domains and a DHHC zinc-finger motif), which are evolutionally conserved from yeasts to the human species (Additional file 5). This raises two questions: (1) is zebrafish Zisp a PAT and, if so, is Noggin-1 a substrate for palmitoylation by Zisp; and (2) what is the biological relevance of Noggin-1 palmitoylation? To answer the first question, RPE1 cells were co-transfected with HA-tagged Noggin-1 and GFP-tagged Zisp. We found a high degree of co-localization between these two proteins (Fig. 7c). Next, we examined the level of palmitoylation using the ABE assay. We found that Noggin-1 was palmitoylated by wild-type Zisp (Fig. 7c, lanes 1 and 2). However, the ability of a mutant form of Zisp (lacking the DHHC domain) to palmitoylate Noggin-1 was greatly reduced (Fig. 7c, lane 6).

In zebrafish, Noggin-1 has two potential palmitoylation sites near the C-terminus (Fig. 7b), which are conserved across species. To investigate which of the two Noggin-1 cytoplasmic cysteine residues can be palmitoylated, we mutated them to alanine residues. Mutation of the cysteine residues in various combinations revealed that not only did Zisp mediated palmitoylation most prominently at Cys-212 (*p* < 0.001) but also at Cys-214 (*p* < 0.0037). Mutation of both Cys-212 and Cys-214 led to complete inhibition of palmitoylation (*p* < 0.001; Fig. 7c, lanes 3–5 and 7d). Therefore, we concluded that zebrafish Noggin-1 is palmitoylated on Cys-212 and/or Cys-214.

Notably, we found that Noggin-1 was mainly distributed in cell–cell junctions in control retinas (Fig. 7e) and within the cytoplasm in the retinas of propofol-treated embryos. Thus, propofol might impair the development of retinogenesis via direct regulation of Noggin-1 secretion and trafficking.

To investigate the biological effect of propofol on Noggin-1, we treated HA-tagged Noggin-1–transfected RPE1 cells with propofol and investigated Noggin-1 secretion and subcellular distribution (Fig. 7f). Propofol reduced the levels of secreted Noggin-1 in a time-dependent fashion (Fig. 7f, lanes 1–5). Soluble intracellular Noggin-1 was increased at low propofol doses (Fig. 7f, lanes 1 and 2) but reduced at high propofol doses (Fig. 7f, lanes 3–5). Surprisingly, when we added the proteasome inhibitor MG132, the levels of both total and soluble intracellular Noggin-1 did not change in response to propofol (Fig. 7g), indicating that upon treatment with propofol, the intracellular pool of Noggin-1 moves into the Triton-soluble fraction, which is readily degraded in a proteasome inhibitor-dependent fashion. Taken together, these results led us to raise the hypothesis that Noggin-1 palmitoylation might be required to stabilize a membrane-associated form of Noggin-1 (i.e., the Triton-insoluble fraction) that may serve as a precursor for secreted Noggin-1 and that propofol might impair this stabilization.

To test this hypothesis, we first investigated the effect of reducing or increasing Zisp levels on Noggin-1 secretion into the media. In RPE1 cells transfected with *nog1* alone, Noggin-1 protein was efficiently secreted, and Noggin-1 could be detected in both Triton-soluble and -insoluble fractions (Fig. 7h, lane 2). When *zisp* was co-transfected with *nog1*, the levels of secreted Noggin-1 increased (Fig. 7h, lane 3). However, co-treatment with propofol reduced Noggin-1 secretion and the amount of Triton-insoluble Noggin-1, while not visibly affecting the levels of soluble intracellular Noggin-1 (Fig. 7h, lane 4).

Next, we co-expressed mutant forms of *nog1* with *zisp* in COS cells and determined the amount of secreted Noggin-1 (Fig. 7i). Although C212A, C214A, and C212A/C214A mutations resulted in only a slight change in baseline Noggin-1 secretion (Fig. 7i, lanes 1, 3, 5, and 7), they reversed the increase in Noggin-1 secretion induced by co-transfection with Zisp (Fig. 7i, lanes 2, 4, 6, and 8). In addition, we found that the level of soluble intracellular Noggin-1 was higher in cells expressing the mutant forms of *nog1* (Fig. 7i, lanes 1, 3, 5, and 7). Meanwhile, in cells expressing the C212A/C214A mutant, the amount of Triton-insoluble Noggin-1 was significantly reduced (Fig. 7i, lanes 7 and 8).

Taken together, these results suggest the dual effect of Zisp; it promotes wild-type Noggin-1 secretion and stabilizes membrane-associated Noggin-1. Propofol inhibits Zisp expression and Noggin-1 trafficking.

## Discussion

Here, we demonstrated the effect of propofol on neuronal development in zebrafish and provided compelling evidence that propofol inhibited the expression of a PAT, Zisp, that controls retinal laminar organization. Our in vivo findings also firstly establish the association between an anesthetic agent and protein palmitoylation in the development of the vertebrate nervous system. This information is useful for further research on the mechanisms via which the improper use of an anesthetic agent might lead to human neurodegenerative diseases.

Propofol is an anesthetic agent, and many studies have demonstrated that it could exert a negative effect on neural stem cell development, ultimately leading to cell death. Our data revealed that in propofol-treated zebrafish embryos, the specification of retinal cell types was inhibited. Most retinoblasts exited the cell cycle too early, i.e., cell cycle progression was compromised. Recent studies have demonstrated that bHLH- and homeobox-type transcription factors contribute to the intrinsic properties of retinal precursors and regulate cell type specification and differentiation [41–43]. For example, the bHLH gene NeuroD and the homeobox gene Crx control photoreceptor generation, and NeuroD and Pax regulate amacrine cells [44]. In propofol-treated embryos, the expression of both *neurod4* and *crx* was reduced, suggesting that propofol inhibited the specification of retinal cell types via regulation of bHLH and homeobox genes. This study is the first to demonstrate the negative effect of propofol in neuronal development signals.

Furthermore, we showed that propofol impaired the specification of retinal cell types via inhibiting Zisp expression. Zisp is a PAT, and Noggin-1 is a specific substrate of Zisp. However, the substrate specificity between Zisp and Noggin-1 remains unclear. One possibility is that the subcellular localization of Zisp plays a role in substrate specificity. Palmitoylation occurs at many cell sites, including the cytosol, plasma membrane, Golgi apparatus/endoplasmic reticulum, and synaptic membranes [45]. Zisp is mainly located in the Golgi apparatus (Additional file 6). Palmitoylation of Noggin-1 by Zisp enhances the association of Noggin-1 with the perinuclear membranous compartment, including Golgi and vesiculotular structures. Thus, Noggin-1 acylation by Zisp might occur in this compartment. A second possibility is that the palmitoylated sequence of Noggin-1 favors the stable interaction of Zisp with Noggin-1. In contrast to other lipid modifications such as myristoylation and prenylation, palmitoylated sequences lack a conserved consensus sequence and can be found at the N- or C-terminus [19]. Noggin-1 was identified as an endogenous substrate, and the palmitoylated sites were found at the C-terminus. However, we revealed the specificity of the interaction between Zisp and Noggin-1 by co-immunoprecipitation. This stable interaction of enzyme and substrate was palmitoylation-dependent because mutations of palmitoylated cysteines abolished the interaction.

Palmitoylation of Noggin-1 serves multiple roles. On the one hand, it affects protein stability. Our data support the hypothesis that palmitoylation was required to tag Noggin-1 to the Golgi membrane, thereby protecting it from proteasome-dependent degradation prior to reaching the cell surface, where it may be rapidly liberated into the extracellular space. A recent report showed that palmitoylation-deficient mutants of the anthrax toxin receptor, tumor endothelial marker 8, undergo ubiquitination, which targets the proteins for degradation in the lysosomes of mammalian cells [46]. Thus, palmitoylation likely regulates protein stability at multiple levels, depending on the localization of each PAT. On the other hand, palmitoylation of Noggin-1 affects protein secretion and signaling activity. Although *nog1* transcripts are expressed in the posterior eye field during somitogenesis [40], Zisp deletion resulted in alterations in laminar organization through inhibition of Noggin-1 trafficking. Therefore, Noggin-1 activity is at moderate or long range during retinogenesis, similar to the positive regulatory role of palmitoylation on Wnt or Chording secretion and activity, rather than to the negative effect of the epidermal growth factor receptor ligand, Spitz [47]. In the case of Spitz, palmitate increases anchorage to the cell membrane, thereby restricting Spitz diffusion and spread [47, 48]. Thus, palmitoylation plays a role in promoting ligand activity at differing ranges.

## Conclusions

We report that propofol, an anesthetic agent, altered the specification of retinal cell types. Our findings establish a direct link between propofol and the regulation of neuronal development, suggesting that the improper use of propofol might result in defects in the neuronal system. Our model could be useful for further research on the mechanisms via which the improper use of anesthetic agents might lead to disease.

## Supplementary Information

**Additional file 1.** Control larvae first respond to paramecia with convergent eye movements, and a high vergence angle is maintained for the duration of the hunting routine at 6 d postfertilization.

**Additional file 2.** Propofol-treated larvae (5 μg/ml propofol) exhibit abnormal behavior and lack convergent eye movements in response to paramecia at 6 d postfertilization.

**Additional file 3. **Propofol increases cell death and decreases the number of S-phase cells in retinas at 36 and 72 h postfertilization (hpf). (a) The number of terminal deoxynucleotidyl transferase dUTP nick-end labeling-positive cells (green) in the retinas of propofol-treated embryos (5 μg/ml propofol) at 36 and 72 hpf is increased compared with that in controls. DNA, blue. Scale bar, 25 μm. (b) 5-Ethynyl-2′-deoxyuridine exposure at 36 and 72 hpf decreases the proportion and mislocalization of S-phase cells in the retinas of propofol-treated embryos (5 μg/ml propofol) compared with control retinas. Error bars indicate the standard error of the mean. *, *p* < 0.05 and **, *p* < 0.01. Scale bar, 18 μm.

**Additional file 4. **Expression of the *zisp* gene during embryogenesis. (a–g) Whole-mount in-situ hybridization of *zisp* at 10, 16, 24, 32, and 52 h postfertilization (hpf). (a, c, and e–g) Lateral views. (b and d) Dorsal views. (h) Temporal expression profiles of *zisp* from the eight-cell stage to 3 dpf were determined by reverse transcription polymerase chain reaction (RT-PCR). *ef1α* was used as an RT-PCR control. Error bars indicate the standard error of the mean.

**Additional file 5. **Amino acid sequence of the Zisp protein. (a) The predicted Zisp domain structure is shown for the transmembrane domain and the Asp-His-His-Cys (DHHC) domain (blue). The DHHC domain of Zisp in zebrafish shows a high degree of conservation with other species. (**b**) Hydropathy was calculated by Hphob (Kyte–Doolittle scale). Hydrophobic regions that are likely to span membranes are denoted by bars.

**Additional file 6.** Noggin-1 co-localized with Zisp in the Golgi apparatus. (a, b) Subcellular localization of Zisp (green) in COS cells. The Golgi apparatus (GM130, red), endoplasmic reticulum (Calnexin, red), and mitochondria (Tom20, red). Zisp primarily localized to the Golgi apparatus (a), and a mutant form of Zisp lacking the Asp-His-His-Cys motif within the cytoplasm (b). (c) Zisp co-localized with Noggin-1 into a perinuclear region of COS cells. Scale bar, 10 μm.

**Additional file 7.** Average and statistical information for Figs. 6 D and I and 7 A, C, E, F, G, H, and I.

## Data Availability

All data generated or analyzed during this study are included in this published article.

## Abbreviations

ABE: Acyl–biotin exchange
bHLH: Basic helix-loop-helix
BMP: Bone morphogenetic protein
DHHC: Asp-His-His-Cys
EdU: 5-Ethynyl-2′-deoxyuridine
GCL: Ganglion cell layer
GFP: Green fluorescent protein
HA: Hemagglutinin
INL: Inner nuclear layer
NaCl: Sodium chloride
ONL: Outer nuclear layer
PAT: Palmitoyl acyltransferase
qRT-PCR: Quantitative reverse transcription polymerase chain reaction
TUNEL: Terminal deoxynucleotidyl transferase dUTP nick-end labeling
