# Supplementary material for: Propofol impairs specification of retinal cell types in zebrafish by inhibiting Zisp-mediated Noggin-1 palmitoylation and trafficking
Source: Stem Cell Res Ther. 2021 Mar 20;12:195. doi: 10.1186/s13287-021-02204-0 (PMC7980560; doi:10.1186/s13287-021-02204-0)
Supplement: Supplementary file 3 — Additional file 3. Propofol increases cell death and decreases the number of S-phase cells in retinas at 36 and 72 h postfertilization (hpf). (a) The number of terminal deoxynucleotidyl transferase dUTP nick-end labeling-positive cells (green) in the retinas of propofol-treated embryos (5 μg/ml propofol) at 36 and 72 hpf is increased compared with that in controls. DNA, blue. Scale bar, 25 μm. (b) 5-Ethynyl-2′-deoxyuridine exposure at 36 and 72 hpf decreases the proportion and mislocalization of S-phase cells in the retinas of propofol-treated embryos (5 μg/ml propofol) compared with control retinas. Error bars indicate the standard error of the mean. *, p < 0.05 and **, p < 0.01. Scale bar, 18 μm. [file 13287_2021_2204_MOESM3_ESM.pdf]

Additional file 3.

File format

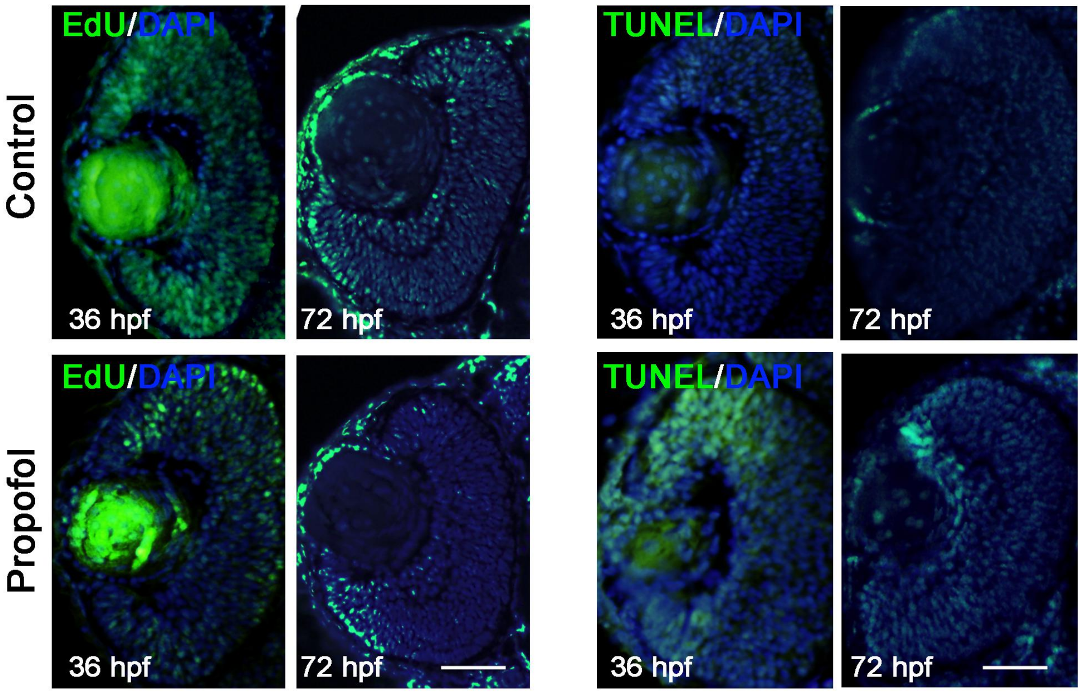

Propofol increases cell death and decreases the number of S-phase cells in retinas at 36 and 72 h postfertilization (hpf). (a) The number of terminal deoxynucleotidyl transferase dUTP nick-end labeling-positive cells (green) in the retinas of propofol-treated embryos (5 µg/ml propofol) at 36 and 72 hpf is increased compared with that in controls. DNA, blue. Scale bar, 25 µm. **(b)** 5-Ethynyl-2′-deoxyuridine exposure at 36 and 72 hpf decreases the proportion and mislocalization of S-phase cells in the retinas of propofol-treated embryos (5 µg/ml propofol) compared with control retinas. Error bars indicate the standard error of the mean. *, $p < 0.05$ and **, $p < 0.01$. Scale bar, 18 µm.
