# Supplementary figures and images for: Propofol impairs specification of retinal cell types in zebrafish by inhibiting Zisp-mediated Noggin-1 palmitoylation and trafficking
Source: Stem Cell Res Ther. 2021 Mar 20;12:195. doi: 10.1186/s13287-021-02204-0 (PMC7980560; doi:10.1186/s13287-021-02204-0)

**Additional file 4.**

**File format**

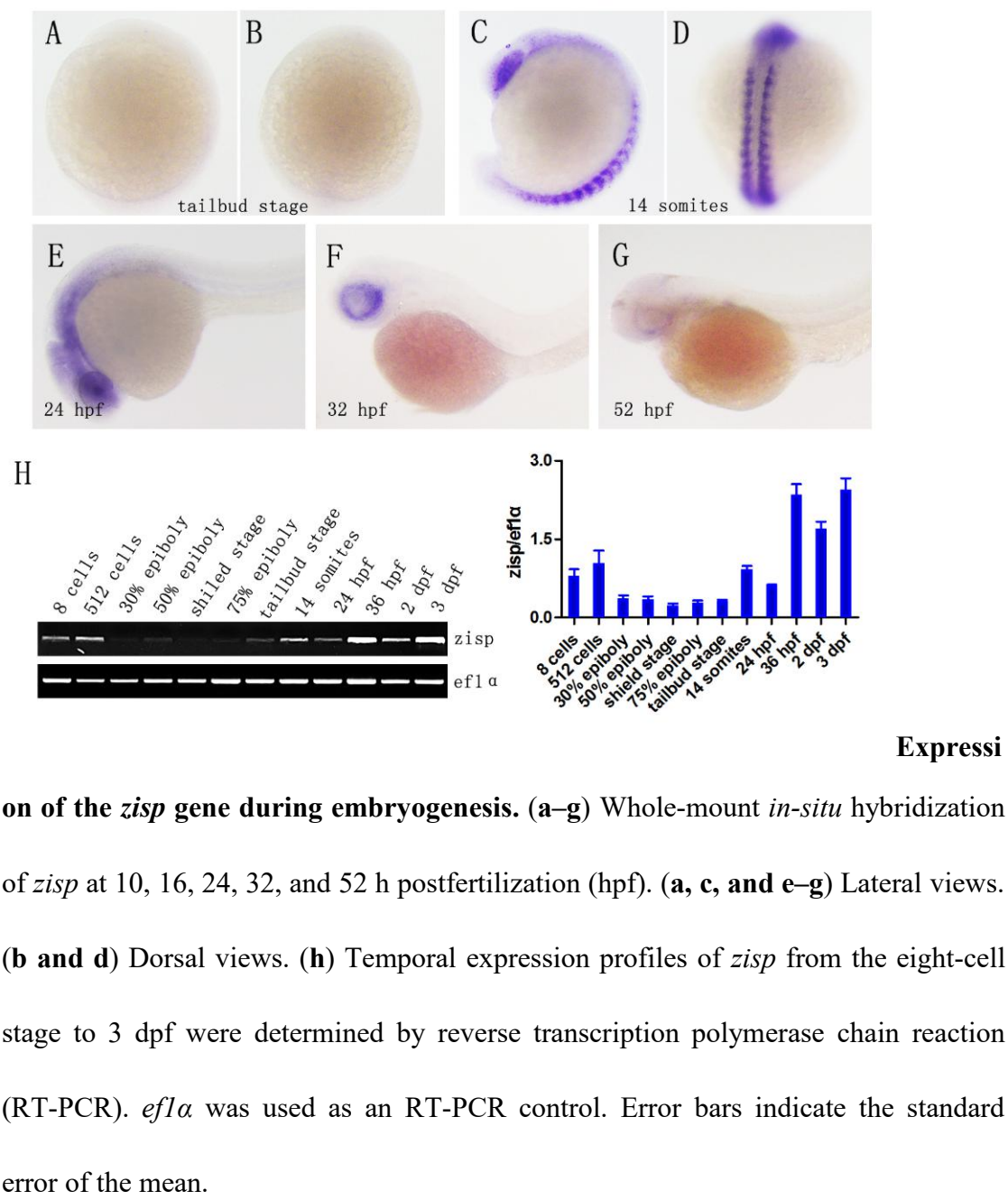

Supplement: Supplementary file 4 — Additional file 4. Expression of the zisp gene during embryogenesis. (a–g) Whole-mount in-situ hybridization of zisp at 10, 16, 24, 32, and 52 h postfertilization (hpf). (a, c, and e–g) Lateral views. (b and d) Dorsal views. (h) Temporal expression profiles of zisp from the eight-cell stage to 3 dpf were determined by reverse transcription polymerase chain reaction (RT-PCR). ef1α was used as an RT-PCR control. Error bars indicate the standard error of the mean. [file 13287_2021_2204_MOESM4_ESM.pdf]
