# Supplementary material for: Propofol impairs specification of retinal cell types in zebrafish by inhibiting Zisp-mediated Noggin-1 palmitoylation and trafficking
Source: Stem Cell Res Ther. 2021 Mar 20;12:195. doi: 10.1186/s13287-021-02204-0 (PMC7980560; doi:10.1186/s13287-021-02204-0)
Supplement: Supplementary file 5 — Additional file 5. Amino acid sequence of the Zisp protein. (a) The predicted Zisp domain structure is shown for the transmembrane domain and the Asp-His-His-Cys (DHHC) domain (blue). The DHHC domain of Zisp in zebrafish shows a high degree of conservation with other species. (b) Hydropathy was calculated by Hphob (Kyte–Doolittle scale). Hydrophobic regions that are likely to span membranes are denoted by bars. [file 13287_2021_2204_MOESM5_ESM.pdf]

Additional file 5.

File format

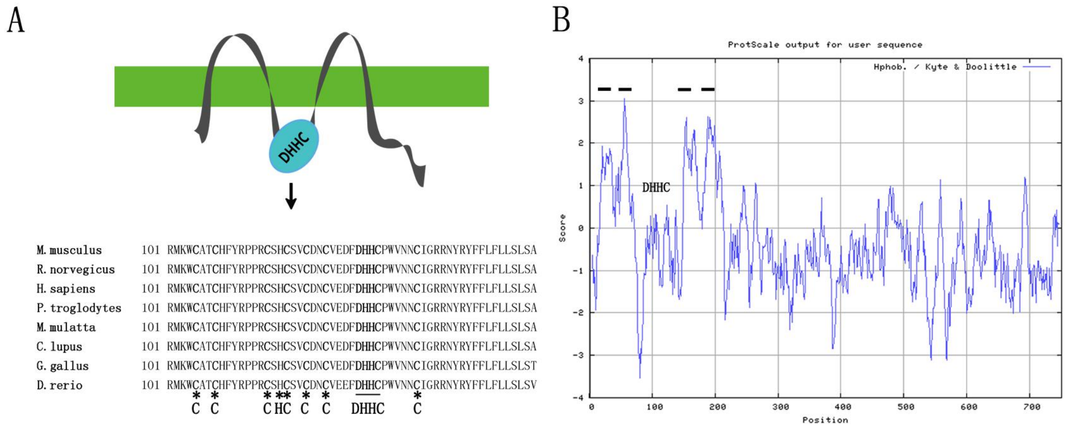

Amino acid sequence of the Zisp protein. (**a**) The predicted Zisp domain structure is shown for the transmembrane domain and the Asp-His-His-Cys (DHHC) domain (blue). The DHHC domain of Zisp in zebrafish shows a high degree of conservation with other species. (**b**) Hydropathy was calculated by Hphob (Kyte–Doolittle scale). Hydrophobic regions that are likely to span membranes are denoted by bars.
