# Supplementary material for: Propofol impairs specification of retinal cell types in zebrafish by inhibiting Zisp-mediated Noggin-1 palmitoylation and trafficking
Source: Stem Cell Res Ther. 2021 Mar 20;12:195. doi: 10.1186/s13287-021-02204-0 (PMC7980560; doi:10.1186/s13287-021-02204-0)
Supplement: Supplementary file 6 — Additional file 6. Noggin-1 co-localized with Zisp in the Golgi apparatus. (a, b) Subcellular localization of Zisp (green) in COS cells. The Golgi apparatus (GM130, red), endoplasmic reticulum (Calnexin, red), and mitochondria (Tom20, red). Zisp primarily localized to the Golgi apparatus (a), and a mutant form of Zisp lacking the Asp-His-His-Cys motif within the cytoplasm (b). (c) Zisp co-localized with Noggin-1 into a perinuclear region of COS cells. Scale bar, 10 μm. [file 13287_2021_2204_MOESM6_ESM.pdf]

Additional file 6.

File format

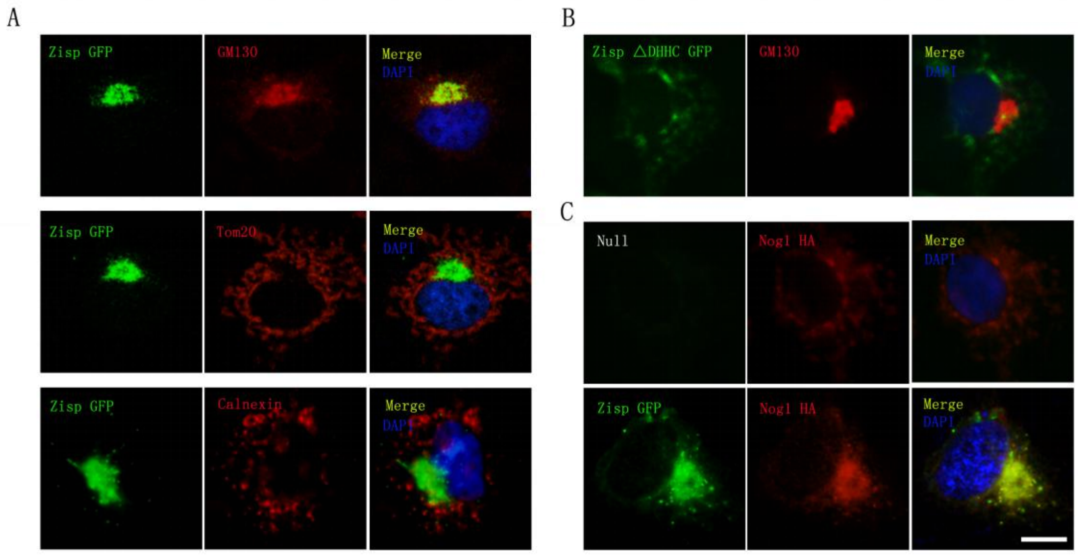

Noggin-1 co-localized with Zisp in the Golgi apparatus. (**a, b**) Subcellular localization of Zisp (green) in COS cells. The Golgi apparatus (GM130, red), endoplasmic reticulum (Calnexin, red), and mitochondria (Tom20, red). Zisp primarily localized to the Golgi apparatus (**a**), and a mutant form of Zisp lacking the Asp-His-His-Cys motif within the cytoplasm (**b**). (**c**) Zisp co-localized with Noggin-1 into a perinuclear region of COS cells. Scale bar, 10 µm.
