# Supplementary material for: Propofol impairs specification of retinal cell types in zebrafish by inhibiting Zisp-mediated Noggin-1 palmitoylation and trafficking
Source: Stem Cell Res Ther. 2021 Mar 20;12:195. doi: 10.1186/s13287-021-02204-0 (PMC7980560; doi:10.1186/s13287-021-02204-0)
Supplement: Supplementary file 7 — Additional file 7. Average and statistical information for Figs. 6 D and I and 7 A, C, E, F, G, H, and I. [file 13287_2021_2204_MOESM7_ESM.pdf]

Additional file 7.

File format

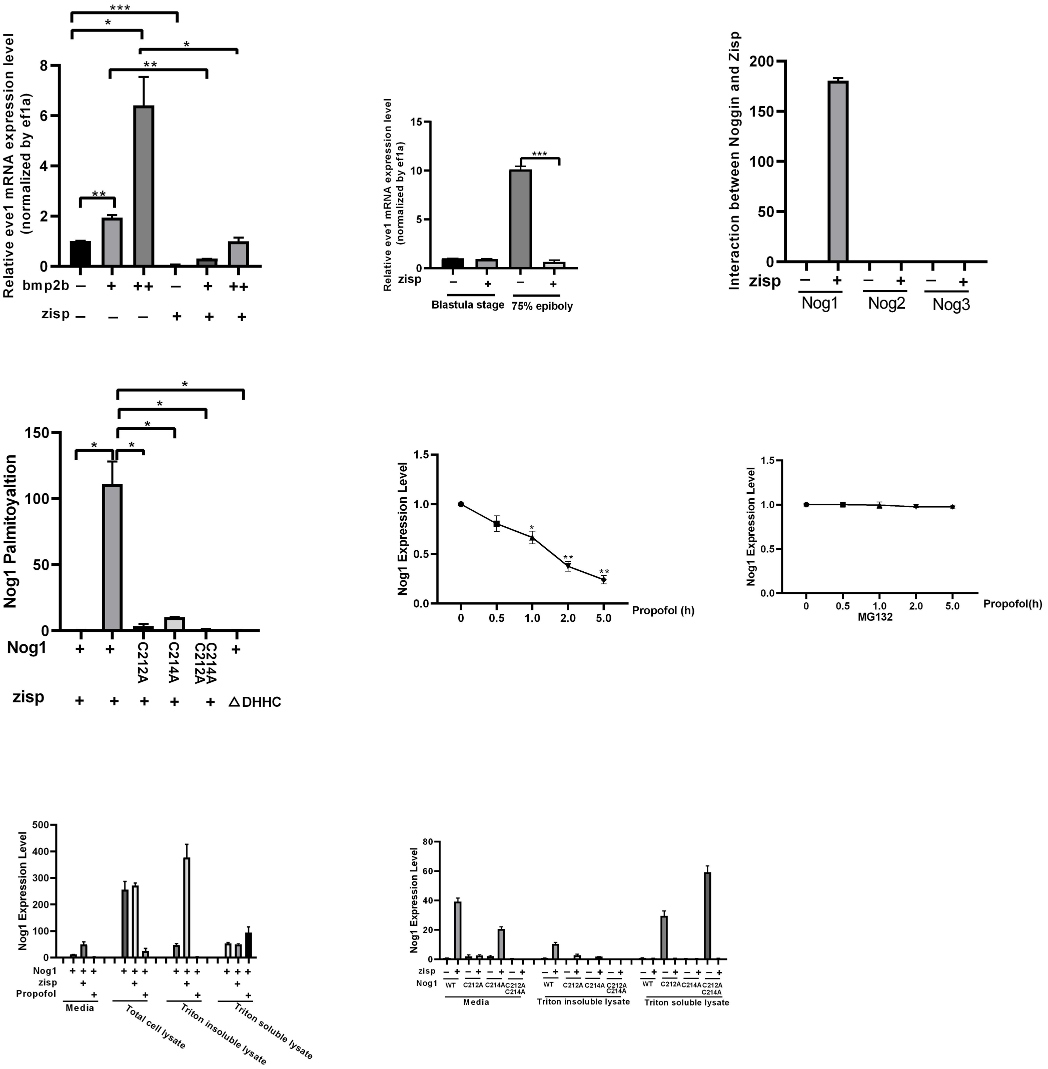

Average and statistical information for Figs. 6 D and I and 7 A, C, E, F, G, H, and I.
